# Knockdown of SNORA47 Inhibits the Tumorigenesis of NSCLC *via* Mediation of PI3K/Akt Signaling Pathway

**DOI:** 10.3389/fonc.2021.620213

**Published:** 2021-03-19

**Authors:** Huiqing Yu, Ling Tian, Liejun Yang, Shihong Liu, Sixiong Wang, Juan Gong

**Affiliations:** Department of Palliative Medicine, Chongqing University Cancer Hospital, Chongqing, China

**Keywords:** NSCLC, SNORA47, EMT, PI3K/AKT, cell growth

## Abstract

**Background:**

Non-small cell lung cancer (NSCLC) is a frequently diagnosed aggressive cancer all over the world. Small nucleolar RNAs (snoRNAs) are a group of non-coding mediatory RNAs. A previous report indicated that small nucleolar RNA 47 (SNORA47) is upregulated in NSCLC. However, the role of SNORA47 in NSCLC is unclear.

**Material and Methods:**

Cell proliferation was measured by immunofluorescence staining. Cell apoptosis and cycle of NSCLC were tested by flow cytometry and the protein expressions were investigated by Western-blot. Meanwhile, cell migration and invasion were detected by transwell assay. Xenograft mice model was established to detect the effect of SNORA47 knockdown on tumor growth of NSLC *in vivo*.

**Results:**

Knockdown of SNORA47 significantly inhibited the proliferation of NSCLC cells *via* inducing cell apoptosis. Moreover, migration and invasion of NSCLC cells were notably decreased by SNORA47 shRNA. SNORA47 knockdown significantly induced G1 arrest in NSCLC cells *via* regulation of p27 Kip1, CDK2, and cyclin D1. Meanwhile, SNORA47 shRNA inhibited EMT process and PI3K/Akt signaling in NSCLC cells. Finally, silencing of SNORA47 significantly inhibited the tumor growth of NSCLC *in vivo*.

**Conclusion:**

Knockdown of SNORA47 significantly inhibited the tumorigenesis of NSCLC *via* inhibition of PI3K/Akt signaling and EMT process. Thereby, our finding might shed a new light on exploring the strategies for the treatment of NSCLC.

## Introduction

Non-small cell lung cancer (NSCLC) is the most frequent type of lung cancer which is known as the leading cause of cancer-associated death all over the world (1). Nowadays, surgery, radiation and chemotherapy are the main treatments of NSCLC (2). Although many efforts have been made to treat NSCLC in the past twenty years, 5-year survival rates of patients with NSCLC remain low due to metastasis and recurrence of lung cancer (3). Therefore, it is necessary to explore new strategies for the treatment of NSCLC.

Small nucleolar RNAs (snoRNAs) are a group of non-coding mediatory RNAs about the length of 60–300 nucleotides which are located in nucleolus of cells (4). It has been previously reported that more than 100 snoRNAs throughout the human body are closely correlated with cellular processes, genesis of miRNA and stress response (5). Since snoRNAs are participated in many physiological processes, dysregulation of snoRNAs may lead to the progression of diseases (6). In addition, it has been confirmed that snoRNAs can act as important mediators in NSCLC. For example, Zheng D et al. found that SNORA78 could promote the tumorigenesis of NSCLC *in vitro* and *in vivo* (7). In addition, Mei YP et al. indicated that SNORA42 could act as an oncogene in NSCLC (8). Meanwhile, SNORA47 is known to be upregulated in NSCLC (9). However, the role of SNORA47 in NSCLC remains unclear.

In the current study, we sought to detect the function of SNORA47 in NSCLC. We hope our research would shed new lights on exploring the new methods for the treatment of NSCLC.

## Material and Methods

### Cell Culture

Human NSCLC cell lines (A549 and NCI-H23), Human normal lung epithelial cells (BEAS-2B) and 293T cells were obtained from American Type Culture Collection (ATCC, Manassas, VA, USA). Cells were maintained in DMEM medium (Thermo Fisher Scientific, Cambridge, MA, USA) supplemented with 10% FBS, penicillin (100 U/ml), and streptomycin (100 μg/ml). Cells were incubated at 37°C with the condition of 5% CO_2_.

### Cell Transfection

SNORA47 shRNA1 or shRNA2 (GenePharma, Shanghai, China) was packaged into lentiviral vector (pLVX-IRES-Puro, GenePharma), and then the lentiviral vector DNAs were transfected into 293T cells (5 × 10^6^/well) including lenti-LINC01234 shRNAs and negative control (NC). After transfection, the cells were incubated at 37°C, and the supernatant was collected. After that, supernatants of two SNORA47 shRNAs and negative control (NC) were filtered into particles. Finally, NSCLC or BEAS-2B cells were infected with lentiviral particles according to the manufactures’ protocol. After 48 h of incubation, stable NSCLC cells were then selected by puromycin (2.5 μg/ml, Sigma Aldrich, St. Louis, MO, USA). The sequences of shRNAs were as follows: SNORA47 shRNA1: 5’-GGACTGAGAAGGTGAGGCAGTTT TTCAAGAGAAAACTGCCTCACCTTCTCAGTCCTTTTTT-3’; SNORA47 shRNA2: 5’-CCTTCCACCGGTTAAGACCTCCATTCAAGAGATGGAGGTCTTAACCGGTGGAAGGTTTTTT-3’; NC shRNA: 5’-CAGCGCTGTGCTGGCCGACATTTTTCAAGAGAAAATGTCGGCCAGCACAGCGCTGTTTTTT.

### Reagents

TGF-β, Akt inhibitor (LY294002), and Erk (PD98059) were purchased from MedChemExpress (MCE; New Jersey, NJ, USA).

### Reverse Transcription-Quantitative PCR (RT-qPCR)

Total RNA was extracted from cell lines or tissues using TRIzol^®^ reagent (Thermo Fisher Scientific). Total RNA was reverse transcribed into cDNA using the PrimeScript RT reagent kit (ELK Biotechnology, Wuhan, China). Subsequently, RT-qPCR was performed using the SYBR premix Ex Taq II kit (ELK Biotechnology, Wuhan, China). RT-qPCRs were performed three times as follows: 2 min at 94°C, followed by 35 cycles (30 s at 94°C and 45 s at 55°C). The following primer pairs were used for RT-qPCR: SNORA47 forward, 5’-GGAGGACTGAGAAGGTGAGGC-3’ and reverse, 5’-GGCAAGGGGACATCCTCTG-3’; β-actin forward, 5’-GTCCACCGCAAATGCTTCTA-3’ and reverse, 5’-TGCTGTCACCTTCACCGTTC-3’. Gene expressions were calculated by 2^−ΔΔt^ method.

### Cell Counting Kit-8 (CCK-8) Assay

NSCLC cells (5 x 10^3^ cells/well) or BEAS-2B cells (5 x 10^3^ cells/well) were plated into 96-well plates and treated with NC or SNORA47 shRNA1 for 0, 24, 48 or 72 h. CCK-8 reagents (10 μl, Beyotime, Shanghai, China) were added into each well, and the plate was then incubated for 2 h at 37°C. The absorbance was detected at 450 nm using a microplate reader (Bio-Rad, Hercules, CA, USA).

### Western Blotting

NSCLC cells were lysed in RIPA lysis buffer (KeyGEN, Nanjing, China), and the concentration of protein was detected by BCA Assay kit (Solar life science, Beijing, China). Sodium dodecyl sulfate-polyacrylamide gel electrophoresis (SDS-PAGE, 10%) was performed to separate the equal amounts of protein (30 μg), and proteins were then shifted onto polyvinylidene difluoride membrane (PVDF, Thermo Fisher Scientific). Five percent nonfat dried milk in TBST was applied to block the PVDF membrane for 1 h. PVDF membrane was incubated at 4°C overnight with the primary antibodies: E-cadherin (Abcam, Cambridge, MA, USA, 1:1000), N-cadherin (Abcam, 1:1000), p-Akt (Abcam, 1:1000), Akt (Abcam, 1:1,000), p-ERK (Abcam, 1:1000), ERK (Abcam, 1:1000), cleaved caspase 3 (Abcam, 1:1000), p27 Kip1 (Abcam, 1:1000), cyclin D1 (Abcam, 1:1000), CDK2 (Abcam, 1:1000) and β-actin (Abcam, 1:1000). Then, the membranes were incubated with HRP-conjugated secondary antibodies (Abcam; 1:5000) for 1 h. Protein bands were visualized using the ECL kit (Thermo Fisher Scientific). β-actin was used as the loading control. IPP 6.0 (Image-Pro Plus 6.0) was used for the densitometry analysis.

### Immunofluorescence Staining

Cells were plated onto a 96-well plate at the density of 5.0 × 10^3^ cells/well. After incubation, cells were transfected with NC or SNORA47 shRNA1 for 72 h. After that, cells were fixed with 4% paraformaldehyde and incubated with rabbit monoclonal antibody anti-Ki67 (1:100, Abcam Cambridge, MA, USA) at 4°C overnight. Then, cells were incubated with anti-rabbit IgG secondary antibody (1:1000, Abcam) for 1 h at room temperature. Finally, the results were observed under a fluorescence microscope (Olympus BX53, Tokyo, Japan).

### Flow Cytometry

Cells were trypsinized and resuspended in binding buffer. After that, cells were stained with 5 µl annexin V-FITC and PI in the dark at 37°C for 30 min. Later on, flow cytometry (FACScan™; BD Biosciences, Franklin Lake, NJ, USA) was applied to analyze the apoptosis rate using CellQuest™ software.

### Cell Cycle Detection

Cell cycle distribution was investigated according to a recent report (10). NSCLC cells were harvested, fixed, permeabilized, and stained with Pharmingen PI/RNase (BD). After incubation for 15 min, cells were analyzed using ModFit and the data were quantified by FlowJo software (BD).

### Transwell Assay

The upper chamber is pre-treated with 100 μl matrigel (BD Biosciences, Franklin Lake, NJ, USA). Cell migration was tested without Matrigel. Subsequently, transfected NSCLC cells were seeded in upper chambers with serum-free media, while the media in lower chambers was supplemented with 10% serum. After 24 h of incubation, transwell chambers were fixed with 5% glutaraldehyde and stained with 0.1% crystal violet. The migrated and invaded cells were calculated under a microscope.

### Xenograft Mice Model

All animal experiments were approved by the research ethics committee of Chongqing University Cancer Hospital. NSCLC cells were infected for 24 h with a control or SNORA47 shRNA-expressing lentivirus (Genepharma), and then injected subcutaneously (2 × 10^6^ cells/per mouse) into 5-week-old BALB/c nude mice (Chinese Academy of Sciences, Shanghai, China). Tumor volume was calculated every week for 5 weeks according to the equation: (length×width×width)/2. At the end of the study, mice were sacrificed for collection of tumor tissues.

### Immunohistochemical (IHC) Staining

Tissues of mice were fixed, paraffin-embedded, and cut into 5-μm-thick sections. Paraffin sections were deparaffinized and rehydrated. The sections were heated in sodium citrate buffer in a microwave for antigen retrieval, washed with phosphate-buffered saline (PBS) for 5 min (three times) at room temperature, incubated in 3% H_2_O_2_ at room temperature for 25 min. The sections were, washed with PBS for 5 min (three times) and blocked and incubated in goat serum for 30 min. Then, samples were incubated with primary antibody (anti-p-Akt and anti-p-ERK, Abcam) overnight at 4°C. After that, samples were incubated with secondary antibody (HRP-labeled) for 30 min at 37°C. Finally, freshly prepared diaminobenzidine (DAB) was added for color development. The tissues were observed under a microscope.

### Statistical Analysis

Data are presented as the mean ± standard deviation. CCK-8 assay was performed in quintuplicate. RT-qPCR, flow cytometry, Ki67 staining, transwell assay, and western blot were repeated three times. Comparisons were analyzed by the Student’s t-test (between two groups) or one-way ANOVA followed by Tukey’s *post hoc* test (among multiple groups, GraphPad Prism; version 7; GraphPad Software, Inc.). P < 0.05 indicates a statistically significant difference.

## Results

### Knockdown of SNORA47 significantly inhibited the proliferation of NSCLC or BEAS-2B cells

**Figure 1:**
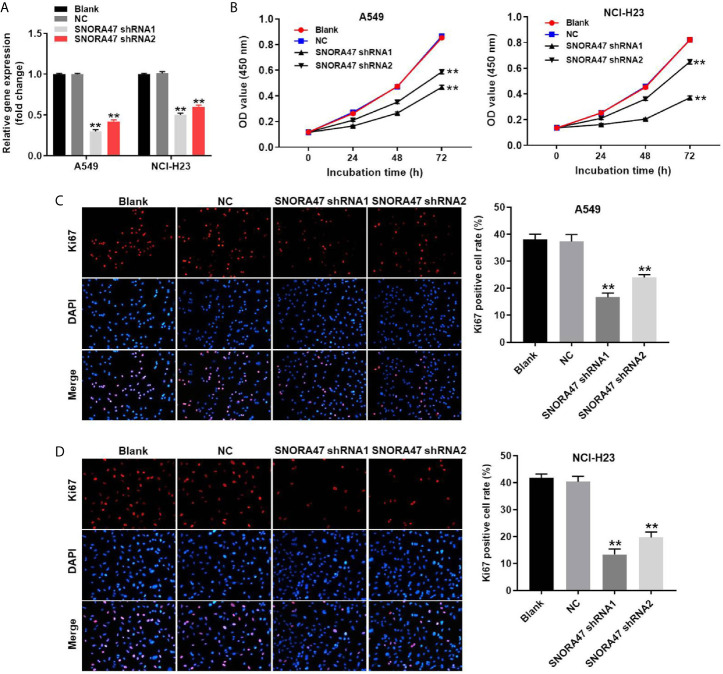
Knockdown of SNORA47 significantly inhibited the proliferation of NSCLC cells. **(A)** A549 or NCI-H23 cells were transfected with SNORA47 shRNA1 or shRNA2. Then, the expression of SNORA47 in NSCLC cells was detected by RT-qPCR. **(B)** The viability of NSCLC cells was tested by CCK-8 assay. **(C)** The proliferation of A549 cells was measured by Ki67 staining. Red fluorescence indicates Ki67. Blue fluorescence indicates DAPI. **(D)** The proliferation of NCI-H23 cells was measured by Ki67 staining. **P < 0.01 compared to Blank.

To detect the efficiency of cell transfection, RT-qPCR was used. As revealed in **Figure 1A** and **Supplementary Figure 1A**, the expression of SNORA47 in NSCLC or BEAS-2B cells was downregulated by SNORA47 shRNA1. Moreover, cell viability of NSCLC or BEAS-2B was notably decreased by SNORA47 knockdown (**Figure 1B** and **Supplementary Figure 1B**), and SNORA47 shRNA1 significantly inhibited the proliferation of NSCLC or BEAS-2B cells (**Figures 1C, D** and **Supplementary Figure 1C**). Meanwhile, the viability and proliferation of BEAS-2B cells were limitedly affected by SNORA47 shRNA2 (**Supplementary Figures 1B, C**). Altogether, knockdown of SNORA47 significantly inhibited the proliferation of NSCLC cells.

### Silencing of SNORA47 Notably Induced the Apoptosis in NSCLC Cells

**Figure 2:**
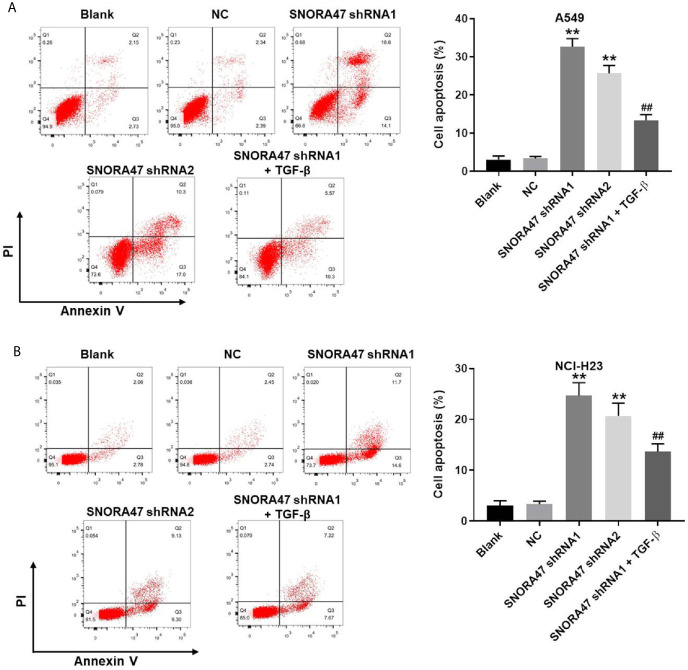
Silencing of SNORA47 notably induced the apoptosis in NSCLC cells. A549 or NCI-H23 cells were treated with NC, SNORA47 shRNA1, SNORA47 shRNA2, or SNORA47 shRNA1 + TGF-β. **(A)** The apoptosis of A549 cells was tested by flow cytometry. **(B)** The apoptosis of NCI-H23 cells was tested by flow cytometry. **P < 0.01 compared to Blank. ^##^P < 0.01 compared to SNORA47 shRNA1.

In order to investigate the effect of SNORA47 knockdown on cell apoptosis, flow cytometry was used. As indicated in **Figures 2A, B** and **Supplementary Figure 1D**, SNORA47 shRNA1 notably induced the apoptosis of NSCLC or BEAS-2B cells, while SNORA47 shRNA2 had very limited effect on BEAS-2B cell apoptosis. Meanwhile, NSCLC cells were more sensitive to SNORA47 shRNA1, compared to BEAS-2B cells (**Figures 1A–D**, **2A, B** and **Supplementary Figures 1A–D**). Furthermore, SNOTRA47 knockdown-induced NSCLC cells apoptosis was partially reversed by TGF-β (**Figures 2A, B**). Thus, silencing of SNORA47 significantly inhibited the proliferation of NSCLC cells *via* inducing cell apoptosis. Moreover, NSCLC cells were more sensitive to SNORA47 shRNA1, compared to SNORA47 shRNA2. Thus, SNORA47 shRNA1 were selected of use in subsequent experiments.

### SNORA47 shRNA1 Significantly Suppressed the Migration and Invasion of NSCLC Cells

**Figure 3:**
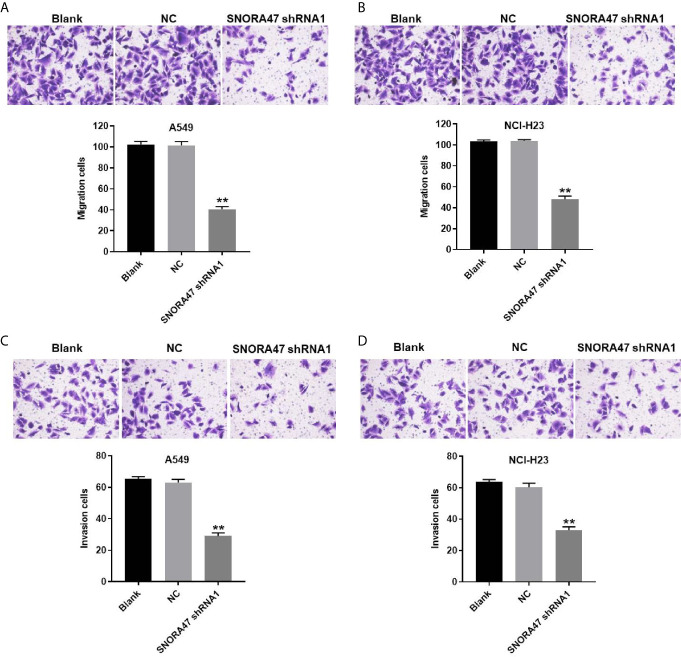
SNORA47 shRNA significantly suppressed the migration and invasion of NSCLC cells. **(A)** The migration of A549 cells was detected by transwell assay. **(B)** The migration of NCI-H23 cells was detected by transwell assay. **(C)** The invasion of A549 cells was detected by transwell assay. **(D)** The invasion of NCI-H23 cells was detected by transwell assay. **P < 0.01 compared to Blank.

In order to detect the migration and invasion of NSCLC cells, transwell was performed. The data showed that migration of NSCLC cells was significantly inhibited by silencing of SNORA47 (**Figures 3A, B**). Consistently, SNORA47 knockdown obviously alleviated the invasion of NSCLC cells (**Figures 3C, D**). Since A549 cells were more susceptible to SNORA47 shRNA treatment, A549 cells were selected for further analysis. Taken together, SNORA47 shRNA significantly suppressed the migration and invasion of NSCLC cells.

### SNORA47 Knockdown Inhibited PI3K/Akt, MAPK/ERK and the EMT Process in NSCLC Cells

**Figure 4:**
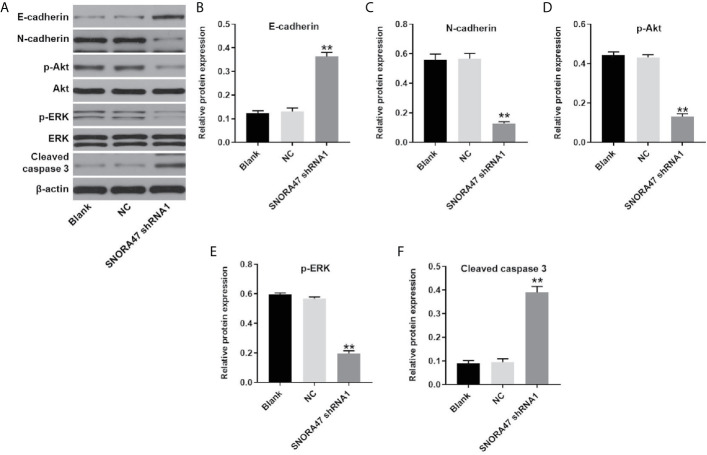
SNORA47 knockdown inhibited PI3K/Akt, MAPK/ERK and the EMT process in NSCLC cells. **(A)** The protein expressions of E-cadherin, N-cadherin, Akt, p-Akt, ERK, p-ERK, and cleaved caspase 3 in NSCLC cells were detected by western blot. **(B–F)** The relative expressions were quantified by normalizing to β-actin. **P < 0.01 compared to Blank.

In order to investigate the mechanism by which SNORA47 mediated the progression of NSCLC, western blot was used. As revealed in **Figures 4A–F**, knockdown of SNORA47 significantly upregulated the expressions of E-cadherin and cleaved caspase 3 and downregulated the expressions of N-cadherin, p-Akt, and p-ERK. In addition, the effect of SNORA47 shRNA1 on E-cadherin and N-cadherin was further increased by LY294002 or PD98059 (**Supplementary Figure 2A**). Altogether, SNORA47 knockdown inhibited PI3K/Akt signaling, MAPK/ERK and the EMT process in NSCLC cells.

### SNORA47 Silencing Notably Induced G1 Arrest in NSCLC Cells

**Figure 5:**
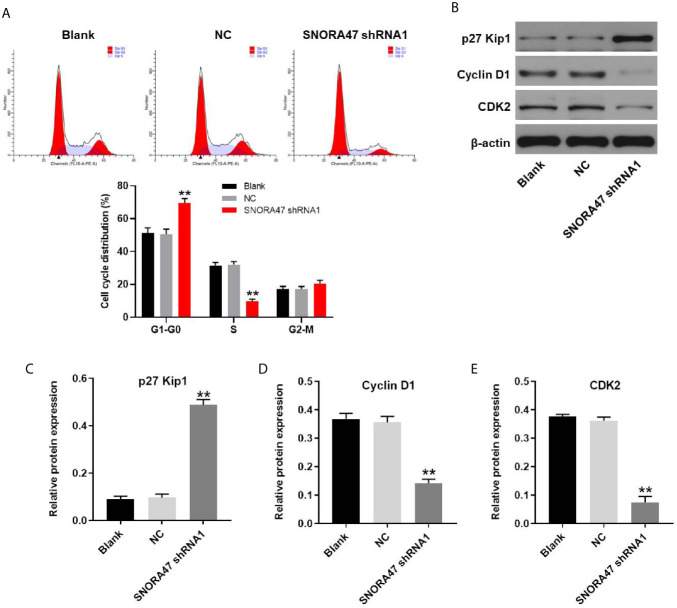
SNORA47 knockdown notably induced G1 arrest in NSCLC cells. **(A)** The cell cycle distribution in NSCLC was detected by flow cytometry. **(B)** The protein expressions of p27 Kip1, Cyclin D1, and CDK2 in NSCLC cells were detected by western blot. **(C–E)** The relative expressions were quantified by normalizing to β-actin. **P < 0.01 compared to Blank.

For the purpose of detecting the effect of SNORA47 knockdown on cell cycle distribution, flow cytometry was performed. As shown in **Figure 5A**, silencing of SNORA47 significantly induced G1 arrest in NSCLC cells. Moreover, the effect of SNORA shRNA1 on cell cycle distribution was further enhanced by LY294002 or PD98059 (**Supplementary Figure 2B**). Meanwhile, the expression of p27 Kip1 in NSCLC cells was notably upregulated by knockdown of SNORA47 (**Figures 5B, C**). In contrast, silencing of SNORA47 significantly inhibited the protein levels of cyclin D1 and CDK2 (**Figures 5B, D, E**). To sum up, SNORA47 knockdown notably induced G1 arrest in NSCLC cells *via* mediation of p27 Kip1, cyclin D1, and CDK2.

### Knockdown of SNORA47 Significantly Inhibited the Tumor Growth of NSCLC *In Vivo*

**Figure 6:**
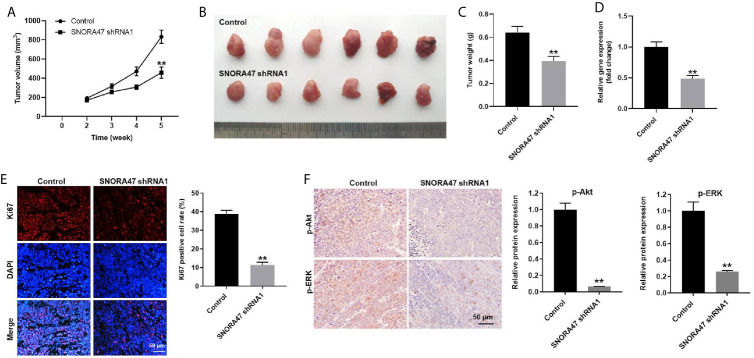
Knockdown of SNORA47 significantly inhibited the tumor growth of NSCLC *in vivo*. A549 cells were subcutaneously injected into nude mice to mimic NSCLC *in vivo*. SNORA47 shRNA1 or vector control was directly injected into the tumors. **(A)** Tumor volumes were assessed once a week. **(B)** Tumor tissues of mice were collected at the end of the study. **(C)** Tumor weights of mice were investigated. **(D)** The level of SNORA47 in mice was investigated by RT-qPCR. **(E)** The proliferation of NSCLC cells was detected by Ki67 staining. **(F)** The protein levels of p-Akt and p-ERK in tissues of mice were measured by IHC staining. **P < 0.01 compared to control.

Finally, xenograft mice model was established to investigate the function of SNORA47 in NSCLC. As revealed in **Figures 6A, B**, the tumor sizes of mice were significantly decreased by knockdown of SNORA47. Consistently, SNORA47 shRNA1 notably reduced the tumor weight of mice (**Figure 6C**). Meanwhile, the level of SNORA47 in mice was significantly decreased when injected with SNORA47 shRNA1 (**Figure 6D**), and SNORA47 shRNA1 notably increased Ki67 positive cell rate in tumor tissue (**Figure 6E**). In addition, the protein levels of p-Akt and p-ERK in tissues of mice were notably inhibited by SNORA47 shRNA1 (**Figure 6F**). In summary, knockdown of SNORA47 significantly inhibited the tumor growth of NSCLC *in vivo*.

## Discussion

In this study, we found that silencing of SNORA47 could lead to the inhibition of cell growth, migration, and invasion in NSCLC. In addition, our research firstly indicated that SNORA47 could play a key role in tumorigenesis of NSCLC *via* mediation of PI3K/Akt/EMT axis. Therefore, our findings would be meaningful for the improvement of new targeted therapy.

It has been reported that snoRNAs are involved in tumorigenesis of NSCLC. For instance, Tang G et al. found that SNORA71A could promote the growth, migration, and invasion of NSCLC cells *via* mediation of MAPK/ERK pathway (11). In addition, Zheng D et al. found SNORA78 could promote the tumorigenesis of NSCLC (7). Moreover, Gao et al. reported that SNORA68 and SNORA21 were notably upregulated in NSCLC tissues, compared with adjacent normal tissues (12). Since these snoRNAs have been confirmed to be participated in the progression of NSCLC, it is necessary to compare the underlying mechanisms of these snoRNAs in NSCLC with SNORA47 for identifying a potential therapeutic target.

PI3K/Akt signaling pathway is frequently upregulated in malignant tumors (13, 14). In the current research, SNORA47 shRNA notably inhibit PI3K/Akt signaling in NSCLC. A recent study found that inactivation of PI3K/Akt signaling contributes to cell apoptosis in NSCLC (15). Thus, our study was consistent to this previous research. Moreover, our data also showed that silencinhg of SNORA47 inactivated p-ERK in NSCLC cells, and ERK inhibitor further increased the apoptotic effect of SNORA47 shRNA. Since ERK is the downstream protein of MAPK pathway (16), our study firstly found the function of SNORA47 in MAPK/ERK signaling. Meanwhile, our findings suggested that knockdown of SNORA47 downregulated EMT process in NSCLC cells. A previous report indicated that activation of PI3K/Akt signaling was correlated with the upregulation of EMT process (17). Taken together with these data, our findings indicated that SNORA47 knockdown could inhibit the progression of NSCLC *via* inhibition of EMT process and PI3K/Akt signaling pathway.

In this research, we found that p27 Kip1, CDK2, and cyclin D1 were the downstream targets of SNORA47 by which SNORA47 shRNA regulated cell cycle distribution of NSCLC. P27 Kip1 is known to be a cell cycle regulator which plays a pivotal role in the G1-to-S transition of the cell cycle (18–20). Consistently, our data demonstrated that SNORA47 knockdown could result in G1 phase arrest in NSCLC cells through upregulation of p27 Kip1. Meanwhile, CDK2 and Cyclin D1 are known to be cell cycle regulators in NSCLC (21–23). SNORA78 could induce cell cycle arrest in NSCLC cells *via* upregulation of CDK2 (7). In addition, SNORA42 could act as an oncogene in lung cancer *via* upregulation of Cyclin D1 (8). Our data was in line with the previous findings of CDK2 and Cyclin D1 as cell-cycle mediators.

According to Li G et al. (24), SNORA47 could promote the tumorigenesis of hepatocellular carcinoma by regulation of EMT process. Similarly, our data also revealed that SNORA47 shRNA could inhibit EMT process in NSCLC cells. In this previous research, SNORA47 significantly inhibited the expression of E-cadherin and increased the level of N-cadherin. Consistently, our study also indicated that SNORA47 silencing inhibited the EMT process *via* regulation of E-cadherin and N-cadherin. Thus, the function of SNORA47 on these two proteins might result in this similarity. Meanwhile, our data further explored the function of SNORA47 in PI3K/Akt signaling.

Of course, there are some limitations in this research as follows: (1) the correlation between SNORA47 and PI3K/Akt signaling is still unclear; (2) other molecular mechanisms by which SNORA47 mediates the tumorigenesis of NSCLC remain to be further explored. Thereby, more investigations are needed in the future.

In conclusion, knockdown of SNORA47 significantly inhibited the tumorigenesis of NSCLC *via* mediation of PI3K/Akt and MAPK/ERK signaling. Thus, SNORA47 might serve as a new target for the treatment of NSCLC.

## Data Availability Statement

The original contributions presented in the study are included in the article/**Supplementary Material**. Further inquiries can be directed to the corresponding author.

## Ethics Statement

The animal study was reviewed and approved by all animal experiments were approved by the research ethics committee of Chongqing University Cancer Hospital.

## Author Contributions

HY and LT conceived and supervised the study. HY, SL, LY, SW, and JG designed and performed the experiments. All authors contributed to the article and approved the submitted version.

## Funding

This study was supported by Chongqing Talents Innovation Leading Talents Program (NO.CQYC201903078) and Key Project of the Climbing Funding of the National Cancer Center(No.NCC201822B74).

## Conflict of Interest

The authors declare that the research was conducted in the absence of any commercial or financial relationships that could be construed as a potential conflict of interest.

## Supplementary Material

Supplementary Figure 1 Knockdown of SNORA47 significantly inhibited the proliferation of BEAS-2B cells *via* inducing apoptosis. **(A)** BEAS-2B cells were transfected with SNORA47 shRNA1 or SNORA47 shRNA2. The expression of SNORA47 in BEAS-2B cells was detected by RT-qPCR. **(B)** The viability of BEAS-2B cells was tested by CCK-8 assay. **(C)** The proliferation of BEAS-2B cells was tested by Ki67 staining. **(D)** The apoptosis of BEAS-2B cells was tested by flow cytometry. **P < 0.01 compared to Blank.

Supplementary Figure 2 The effect of SNORA47 knockdown on cell cycle and EMT process was further enhanced by LY294002 or PD98059. NSCLC cells were treated with NC, SNORA47 shRNA1, SNORA47 shRNA1 + LY294002 or SNORA47 shRNA1 + PD98059. **(A)** The protein levels of E-cadherin and N-cadherin in NSCLC cells were detected by western blot. The relative expressions were quantified by normalizing to β-actin. **(B)** The cell cycle distribution was tested by flow cytometry. **P < 0.01 compared to Blank. ^##^P < 0.01 compared to SNORA47 shRNA1.
