# Supplementary figures and images for: Knockdown of SNORA47 Inhibits the Tumorigenesis of NSCLC via Mediation of PI3K/Akt Signaling Pathway
Source: Front Oncol. 2021 Mar 19;11:620213. doi: 10.3389/fonc.2021.620213 (PMC8017274; doi:10.3389/fonc.2021.620213)

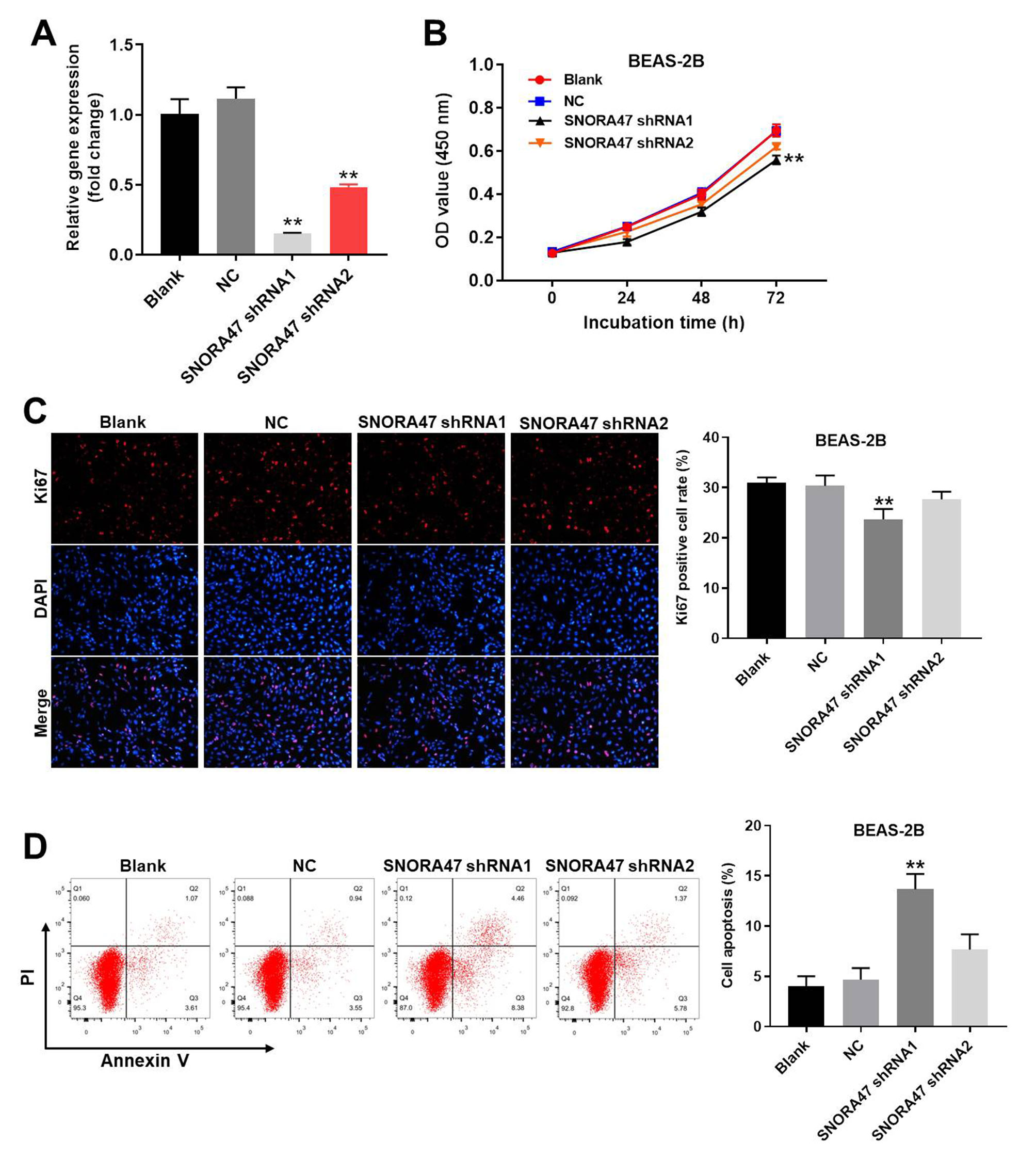

Supplement: Supplementary Figure 1 — Knockdown of SNORA47 significantly inhibited the proliferation of BEAS-2B cells via inducing apoptosis. (A) BEAS-2B cells were transfected with SNORA47 shRNA1 or SNORA47 shRNA2. The expression of SNORA47 in BEAS-2B cells was detected by RT-qPCR. (B) The viability of BEAS-2B cells was tested by CCK-8 assay. (C) The proliferation of BEAS-2B cells was tested by Ki67 staining. (D) The apoptosis of BEAS-2B cells was tested by flow cytometry. **P < 0.01 compared to Blank. [file Image_1.jpeg]

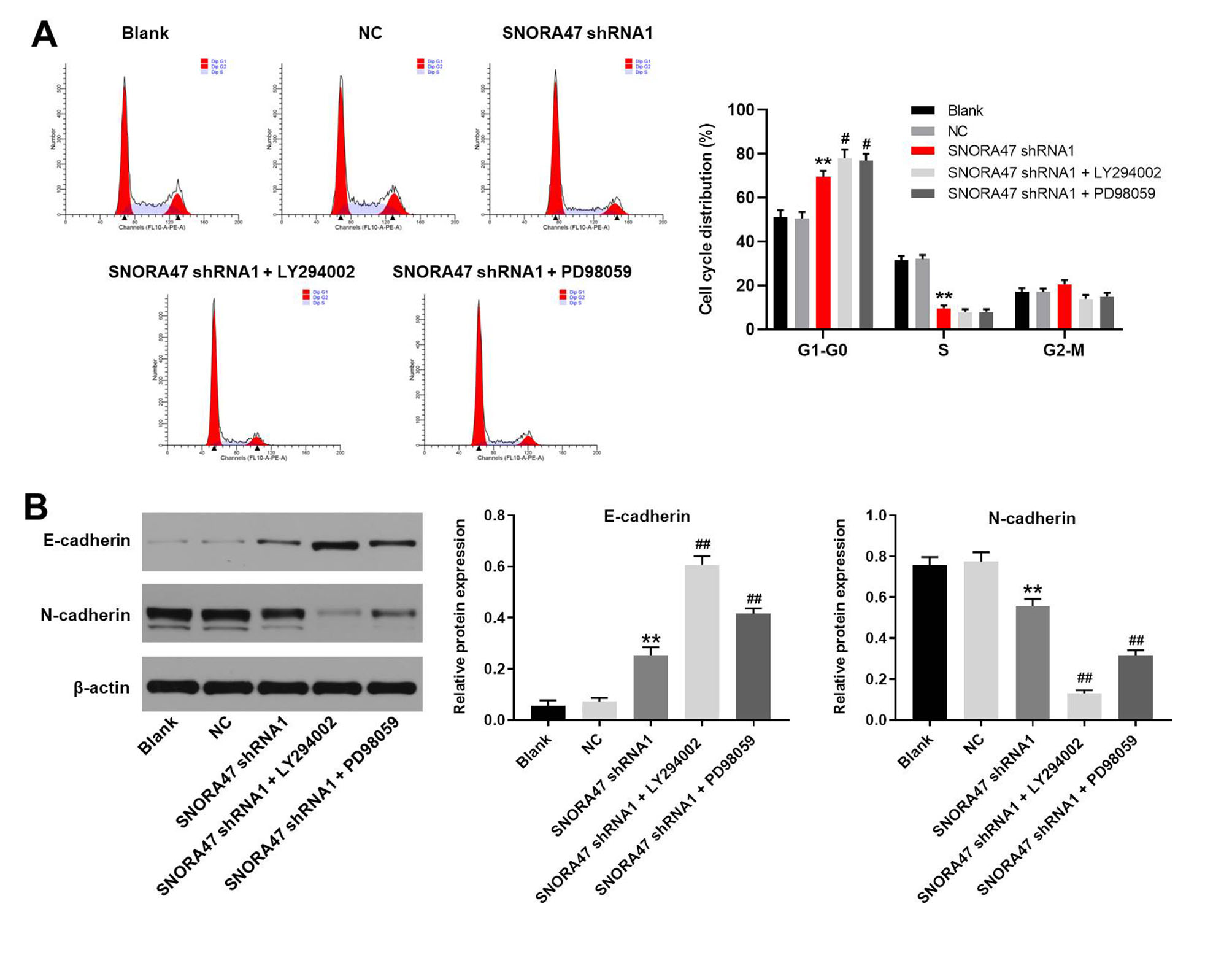

Supplement: Supplementary Figure 2 — The effect of SNORA47 knockdown on cell cycle and EMT process was further enhanced by LY294002 or PD98059. NSCLC cells were treated with NC, SNORA47 shRNA1, SNORA47 shRNA1 + LY294002 or SNORA47 shRNA1 + PD98059. (A) The protein levels of E-cadherin and N-cadherin in NSCLC cells were detected by western blot. The relative expressions were quantified by normalizing to β-actin. (B) The cell cycle distribution was tested by flow cytometry. **P < 0.01 compared to Blank. ##P < 0.01 compared to SNORA47 shRNA1. [file Image_2.jpeg]
